# Predictors of colonoscopy use one year after colonoscopy: prospective study of surveillance behavior for colorectal cancer

**DOI:** 10.1080/21642850.2014.889573

**Published:** 2014-03-06

**Authors:** Toshiatsu Taniguchi, Kei Hirai, Ryoko Sumi, Noriyuki Hayashi, Kazuhisa Maeda, Toshinori Ito

**Affiliations:** ^a^ Department of Psychiatry, Tottori Seikyo Hospital, 458 Suehiroonsenn-cho, Tottori680-0841, Japan; ^b^ Department of Complementary and Alternative Medicine, Osaka University Graduate School of Medicine, 2-2 Yamadaoka, Suita, Osaka565-0871, Japan

**Keywords:** colorectal cancer, colonoscopy, surveillance behavior, cancer threat, prospective study

## Abstract

We hypothesized that perceived risk of colorectal cancer (CRC) and CRC worry would be the main predictors of surveillance behavior in patients undergoing colonoscopy. We therefore assessed factors predicting colonoscopy use for re-examination one year after colonoscopy. Patients who had undergone colonoscopy and were scheduled for re-examination one year later were recruited. Patients were administered questionnaires after baseline colonoscopy assessing demographic factors, perceived risk, CRC worry, cancer preventability, knowledge of CRC and results of colonoscopy. We confirmed whether participants underwent colonoscopy re-examinations one year later (follow-up). Finally, 56 participants completed the research and were used in the final analysis (response rate = 65.1%). We found that 37.5% of the participants who underwent baseline colonoscopy underwent follow-up colonoscopy one year later. Follow-up colonoscopy was not significantly associated with any psychological variables, but was significantly associated with educational status (postsecondary) (odds ratio [OR] = 7.10, 95% confidence interval [CI] = 1.83–27.56) and the results of baseline colonoscopy in patients who did not undergo polypectomy but had remaining polyps (OR = 4.26, 95% CI = 1.02–17.84). Additionally, significant differences in cancer threat-related variables were observed among groups of patients who, during baseline colonoscopy, underwent polypectomy but had no remaining polyps, had polyps removed with some polyps remaining, or did not undergo polypectomy but had remaining polyps (*p* < .05), with the latter group having a significant relationship with repeat colonoscopy. Cancer threat-related variables were not predictive of repeat colonoscopy after one year. In contrast, patient educational status and the colonoscopy results were predictors. We also found a non-linear relationship between high CRC threat and inhibition of the screening behavior in that the CRC threat functions as motivation for the surveillance behavior of colonoscopy.

## Introduction

Incidence rates for colorectal cancer (CRC) have markedly increased worldwide, with substantial changes observed in Japan (Center, Jemal, & Ward, 2009). According to the Ministry of Health, Labour and Welfare, CRC is the leading cause of cancer-related deaths in Japanese women and is associated with increased rates of morbidity (Matsuda et al., 2012). Early detection and treatment of CRC can improve patient prognosis, with fecal occult blood tests (FOBT) and colonoscopy significantly decreasing CRC mortality rates (Baxter et al., 2009; Lee et al., 2007; Rex, Johnson, Lieberman, Burt, & Sonnenberg, 2000; Saito et al., 1995; Smith, Cokkinides, & Eyre, 2006; Zauber et al., 2012).

In Japan, people aged ≥40 years are required to undergo yearly FOBT for CRC screening, and those with positive FOBT results are recommended to undergo colonoscopy (Lee et al., 2007). According to Japanese Society for Cancer of the Colon and Rectum Guidelines (2010), the method of treatment depends on polyp size, depth of invasion, tumor morphology and histological type. Re-examination within three years of colonoscopic polypectomy is recommended to assess recurrence. Studies in US patients with polypectomy-removed adenomas have shown that re-examination within three years is appropriate (Winawer et al., 1993), although most patients in the USA are re-examined at shorter intervals (Lieberman et al., 2012). In Japan, the intervals for re-examination for patients with removed polyps ≥6 mm in size, including those with early colorectal carcinomas confined to the mucosa (m cancer) and submucosa (sm cancer), average about 15 months (Matsuda et al., 2009). However, 24–40% of patients diagnosed with CRC and considered at high risk of recurrence do not undergo follow-up colonoscopy within three years (Cooper & Payes, 2006; Elston Lafata, Cole Johnson, Ben-Menachem, & Morlock, 2001; Laiyemo et al., 2010), suggesting the need to improve colonoscopic surveillance rates.

Predictors of CRC surveillance include demographic characteristics such as age, race, socioeconomic status, and marital status (Elston et al., 2001; Rolnick et al., 2005; Rulyak, Mandelson, Brentnall, Rutter, & Wangner, 2004). Because these factors cannot be controlled externally, effective interventions for behavioral change are limited. Identification of controllable factors, such as psychological factors or knowledge that strongly influences behavior, is important to develop effective measures to improve colonoscopic surveillance rate. The only study to date addressing psychological factors related to CRC surveillance behavior was a cross-sectional study investigating predictors of intention to undergo colonoscopy among CRC survivors (Salz et al., 2009), based on the Health Belief Model (Rosenstock, 1974). The perceived likelihood of CRC was the only factor significantly associated with intention to undergo colonoscopy, whereas perceived benefits, barriers, and self-efficacy were not. Moreover, that study assessed whether patients intended to undergo colonoscopy within five years, differing from the period recommended in clinical settings.

This study was designed to investigate the individual predictors of repeat colonoscopy one year after initial colonoscopy in clinical settings. Factors assessed included cancer threat-related variables, such as perceived risk and CRC worry. Perceived risk was shown to be the main predictor of CRC surveillance (Salz et al., 2009) and of CRC screening (Vernon, 1999). Cancer worry has also been assessed, mainly in relation to breast cancer screening (Lerman et al., 1991; Seki et al., 2011). More importantly, these variables are likely to be observable and modifiable in clinical settings. We therefore hypothesized that both perceived risk and CRC worry would be significantly associated with follow-up colonoscopy one year after initial colonoscopy. Because the preventability of CRC has also been shown to significantly influence CRC screening intention (Codori, Petersen, Miglioretti, & Boyd, 2001), we examined the relationship between CRC preventability and surveillance behavior. Finally, although knowledge of CRC or CRC screening was shown to be associated with CRC screening behavior (Ng, Tan, Teo, Seah, & Phua, 2007), other studies have suggested that CRC knowledge was not a predictor of CRC screening behavior (Manne et al., 2001; Weinberg et al., 2009). Therefore, to clarify this relationship, we examined the association between CRC knowledge and colonoscopy surveillance behavior.

## Methods

### Participants and design

This study was approved by the Institutional Review Board for Clinical Research at Osaka University Hospital. From February 2011 to September 2013, this prospective study was conducted at three general hospitals (A–C). Using a consecutive sampling procedure, we recruited patients who underwent colonoscopy at each hospital and were (1) instructed to undergo follow-up colonoscopy one year later, (2) had no history of abdominal or laparoscopic surgery for CRC, (3) had no history of hereditary non-polyposis colorectal cancer or familial adenomatous polyposis, and (4) understood Japanese. Each selected patient provided written informed consent after receiving their test result and the time of next colonoscopy from their physician. Participants who consented were administered questionnaires, either directly or by mail, within one month after receiving the results of baseline colonoscopy.

Patients were recommended to undergo colonoscopy one year ± three months after baseline colonoscopy. Hospital records were therefore examined to determine whether these patients had undergone follow-up colonoscopy within 15 months of baseline colonoscopy. Moreover, because patients may have undergone follow-up colonoscopy at another hospital, we contacted each patient by telephone or mail to determine whether they had done so. We also determined if these patients had undergone colonoscopy earlier than scheduled owing to other health problems.

### Measures

We developed our own questionnaire to determine predictors for colonoscopic surveillance. Items developed with reference to previous studies in the West were modified based on whether they were applicable in Japan. The questionnaire consisted of four parts.

The first part asked about physician recommendation for follow-up colonoscopy and patient satisfaction with colonoscopy. Previous studies have shown that physician recommendation was the main predictor of CRC screening (Farmer, Bastani, Kwan, Belman, & Ganz, 2008; Wee, McCarthy, & Phillips, 2005) and that satisfaction with the doctor–patient relationship also significantly influenced patient compliance (Haas et al., 2000; Rubin et al., 1993). Participants were asked about how strongly their physicians recommended they undergo follow-up colonoscopy, with answers rated on a five-point Likert scale, from 1 (not recommended) to 5 (strongly recommended). Satisfaction with colonoscopy was assessed using three items (Hass et al., 2000): (1) Would you recommend this procedure to family or friends? (2) Was it painful or not painful? and (3) I was satisfied with the medical explanation and/or treatment. Each of these three items was scored on a five-point Likert scale, from 1 (strongly disagree) to 5 (strongly agree).

The second part of the questionnaire asked about individual variables associated with CRC, including perceived risk, CRC worry, preventability, and CRC knowledge. Perceived risk consisted of six items, including two from previous studies (Manne et al., 2001; Tsubono, Fukao, Hisamichi, Sugawara, & Hosokawa, 1993). The first item was a 0–100 rating of lifetime risk. The other five items consisted of items assessing personal risk of CRC compared with individuals of the same age and/or gender, and perceived seriousness of CRC, each scored from 1 (strongly disagree) to 5 (strongly agree). CRC worry was assessed according to four items developed from a previous study (Seki et al., 2011). These items included worry about undergoing CRC screening, and impact on mood and daily life, each scored from 1 (strongly disagree) to 5 (strongly agree). Perceived preventability was assessed using a single question (Manne et al., 2001), “How much do you think you can prevent CRC?” rated on a scale of 0–100%. CRC knowledge was assessed using 13 items, based on previous studies (Green & Kelly, 2004; Weinberg et al., 2009), including current rates of CRC morbidity and mortality, risk of CRC morbidity, and type of CRC screening tests.

The third and fourth parts of this questionnaire asked about participants' demographic and clinical background and health status, including sex, age, marital status, employment status, economic status, and educational status. Questions about health status included smoking and drinking habits, history of disorders, regularity of visits to hospital, family history of CRC, and previous history of colonoscopy.

Physician reports and medical records were assessed to determine the results of colonoscopy and whether patients underwent follow-up colonoscopy. Colonoscopy results included: (a) whether patients had undergone polypectomy (yes or no), (b) whether a polyp remained after colonoscopy (yes or no), (c) the maximum size of the removed polyp (<5, 5–10, or >10 mm), and (d) the result of pathological examination of the polyp (adenoma, m cancer, or sm cancer). Colonoscopy re-examination was confirmed from medical records and patient report via telephone or mail (method used or unused).

### Statistical analysis

Reliability coefficients were calculated to verify the reliability of measures of risk perception, CRC worry, and satisfaction with colonoscopy. Descriptive analysis was performed to summarize background variables. Initially, univariate analysis (*χ* ^2^ test and Mann–Whitney test) was performed to assess the association of each variable with surveillance behavior. Multiple logistic regression analyses, including variables significant on univariate analysis (*p* < .1), were performed to assess whether each of these variables was independently associated with surveillance behavior. Qualitative variables including demographics, clinical background, health status, and result of colonoscopy were included in each analysis as dummy variables. All analyses were two-tailed, with *p*-values < .05 considered statistically significant. The statistical significance of variables included in multiple logistic regression analyses was determined using likelihood ratio tests. All statistical analyses were performed using SPSS (version 17.0, IBM Corporation, Armonk, NY).

## Results

### Background of participants and status of follow-up colonoscopy

**Figure 1.**
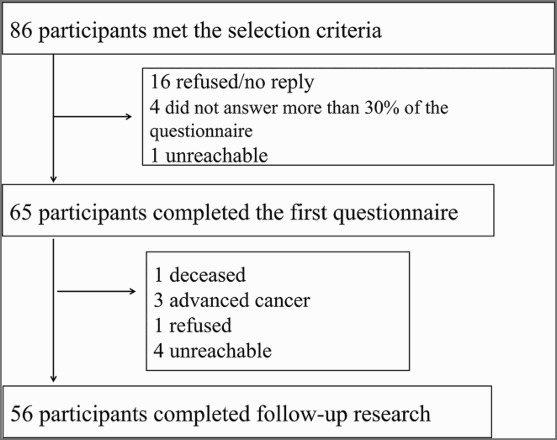
Flow diagram of study participants.

Eighty-six individuals were identified as candidates for this study. At baseline, 21 individuals were excluded, 16 owing to no reply or refusal to participate, 4 who did not fill out more than 30% of their questionnaires, and 1 who was unreachable. At follow-up, nine additional individuals were excluded, including one who died, three who had advanced cancer, one who refused participation, and four who were unreachable. Thus, 56 individuals were included in the analysis (Figure 1), making the response rate 65.1%: 44 cases (79%) from hospital A, 3 cases (5%) from hospital B, and 9 cases (16%) from hospital C. Hospital B discontinued participation during the study, while hospital C joined the study late.

 Table 1 summarizes the demographic and diagnostic characteristics of the study participants. Mean subject age was 64.4 ± 9.6 years. Polyps were removed from 43 of the 56 (76.8%) participants, with 40 having adenomatous polyps and none having either sm cancer or m cancer. Forty-one subjects (73.2%) had polyps remaining after polypectomy. The subjects were also classified into four groups (two groups that underwent polypectomy or not, and two groups that had remaining polyps or not) because the presence or absence of polypectomy was associated with the remaining polyp condition in the colonoscopy results. Thus, of the 56 patients, 15 (28.6%) underwent polypectomy with no remaining polyps, 28 (50%) had polypectomy with some polyps remaining, and 13 (23.2%) did not undergo polypectomy but had remaining polyps. None of the patients who did not undergo polypectomy had no remaining polyps, because all patients had polyps in their colon or rectum.

**Table 1.** Demographic variables across status of colonoscopy rechecking (*n* = 56).

	Total		Unused		Used		*p* (*χ*^2^test)
	*n* = 56	% (=Yes)	*n* = 35	% (Yes)	*n* = 21	% (=Yes)	
Age (years)							
40–65	30	53.6	17	56.7	13	43.3	.33
65–81	26	46.4	18	69.2	8	30.8	
Sex							
Male	41	73.2	23	56.1	18	43.9	.10
Female	15	26.8	12	80.0	3	20.0	
Marital status							
Married	45	80.4	27	60.0	18	40.0	.42
Unmarried	10	17.9	7	70.0	3	30.0	
No information	1	1.8					
Education status							
Up to high school	38	67.9	28	73.7	10	26.3	.005
Postsecondary	16	28.6	5	31.3	11	68.8	
No information	2	3.6					
Employed							
Unemployed	28	50.0	19		9		.25
Employed	27	48.2	15		12		
No	1	1.8					
Household income^a^							
Up to 3000	25	44.6	19		6		.15
3000–7000	18	32.1	9	50.0	9	50.0	
>7000	10	17.9	5	50.0	5		
No information	3	5.4					
Familial history of CRC							
No	39	69.6	24	68.6	15	71.4	.82
Yes	17	30.4	11	31.4	6	28.6	
History of having colonoscopy							
No	28	50.0	18	51.4	10	47.6	.78
Yes	28	50.0	17	48.6	11	52.4	
Maximum size of polyps^b^							
<5 mm	26	46.4	15	57.7	11	42.3	.58
5–10 mm	12	21.2	7	58.3	5	41.7	
>11 mm	18	32.1	13	72.2	5	27.8	
Result of colonoscopy							
Polypectomy/no polyps	15	26.8	12	80.0	3	20.0	.07
Polypectomy/remaining polyp	28	50.0	18	64.3	10	35.7	
Not Polypectomy/remaining polyp	13	23.2	5	38.5	8	61.5	

^a^AMOUNT (UNIT: JPY1000).

^b^These polyps included removed or remaining polyps.

Of the 56 patients who underwent baseline colonoscopy, 21 (37.5%) underwent follow-up colonoscopy one year later (Table 1). Patients who did and did not undergo follow-up colonoscopy differed significantly in educational status (*p* < .01) and in the results of baseline colonoscopy (*p* < .05).

### Psychological factors associated with follow-up colonoscopy after one year

In assessing reliability coefficients, we found that CRC worry (Cronbach's alpha = 0.77) was sufficient for analysis, whereas perceived risk (Cronbach's alpha = −0.01) and satisfaction with colonoscopy (Cronbach's alpha = 0.51) were not. Therefore, perceived risk was analyzed as a single item and satisfaction with colonoscopy as two of the three items: satisfaction with medical explanation or treatment was defined as satisfaction with the physician, and painless colonoscopy was reversed and defined as pain during colonoscopy. Bivariate analysis assessing the association between each psychological factor and follow-up colonoscopy found that physician recommendation was the only factor significantly associated with repeat colonoscopy (*p* < .05; Table 2).

**Table 2.** Comparison of psychological variables and follow-up colonoscopy.

	Unused (*n* = 35)		Used (*n* = 21)		*p*
	Mean	SD	Mean	SD	
Physician recommendation	3.26	1.12	3.90	0.94	.01
Suffering colonoscopy	2.85	1.43	2.90	1.48	.96
Physician satisfaction	4.31	1.11	4.10	1.48	.78
CRC knowledge	6.09	3.364	7.33	2.799	.16
CRC worry	11.86	3.327	11.71	4.268	.83
Perceived preventability (0–100%)	49.71	23.33	55.71	30.09	.28
Perceived risk (0–100%)	43.71	18.80	54.76	28.22	.14
Perceive risk (each item)					
I think the risk of CRC is high	3.06	0.998	3.48	1.078	.16
Compared with average women of the same age, morbidity of CRC is high	3.03	0.954	2.67	1.017	.19
Compared with average men of the same age, morbidity of CRC is low^a^	3.29	0.987	3.19	1.123	.68
Recovery from CRC is easier compared with other diseases^a^	3.14	1.089	2.81	1.209	.21
If I suffer from colon cancer, I will not completely heal from it	2.74	0.852	2.67	0.913	.72

^a^The item's score was reversed before analysis.

### Predictors of repeat colonoscopy after one year

Factors significantly predictive of repeat colonoscopy after one year, including factors associated with educational status, results of baseline colonoscopy, physician recommendation, and psychological variables such as CRC worry, perceived risk (0–100%) and perceived preventability (0–100), were analyzed by multiple logistic regression analysis. Educational status (postsecondary) (odds ratio [OR] = 7.10, 95% confidence interval [CI] = 1.83–27.56) and the results of baseline colonoscopy (not polypectomy/remaining polyps) (OR = 4.26, 95% CI = 1.02–17.84) were the only predictive factors, whereas none of the psychological variables were predictive (Table 3).

**Table 3.** Multiple logistic regression analysis of colonoscopy after one year^a^

Variables	OR	95% CI
CRC worry	–	–
Perceived preventability (0–100%)	–	–
Perceived risk (0–100%)	–	–
Educational status (postsecondary)	7.10	1.83–27.56
Result of colonoscopy (not polypectomy/remaining polyp)	4.26	1.02–17.84

^a^Adjusted: sex, household income, and physician recommendation.

### Psychological factors associated with the results of baseline colonoscopy

**Figure 2.**
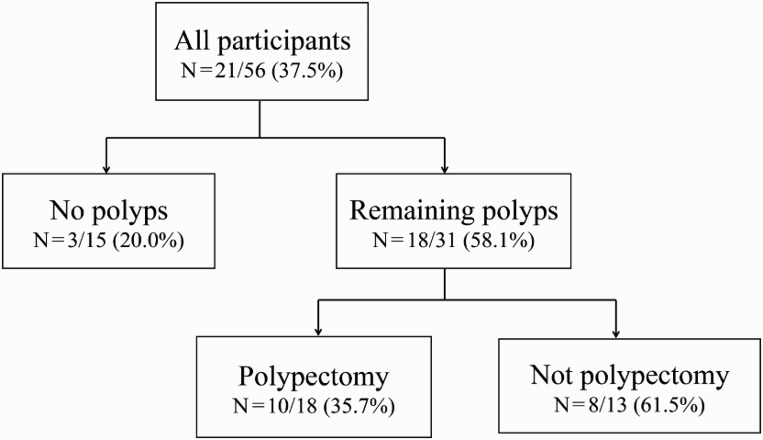
Flow chart showing detection of polyps and their removal on colonoscopy. The numbers in each category are the rate of repeat colonoscopy use one year after initial colonoscopy.

Multivariate analyses suggested that the three categories of baseline colonoscopy results (polypectomy with no remaining polyps, polypectomy with some polyps remaining, and not polypectomy but remaining polyps) were associated with psychological factors (Figure 2). These three patient groups differed significantly in CRC worry (*p* < .05) and in their response to the statement, “CRC heals more easily than other diseases” (*p* < .05) (Table 4). We also used Bonferroni's method to adjust the level of significance and the Mann–Whitney *U*-test to assess differences between the three groups. The result was that for both tests, results for “not polypectomy/remaining polyps” were lower than for “polypectomy/remaining polyps”, and we identified a significant difference (*p* < .05) in CRC worry and marginally significant difference (*p* < .1) in “CRC is easier to cure than other illnesses”.

**Table 4.** Comparison of psychological variables on follow-up colonoscopy.

Variables	Polypectomy/no polyps (*N* = 15)		Polypectomy/remaining polyps (*N* = 28)		Not Polypectomy/remaining polyps (*N* = 13)		*p*
	Mean	SD	Mean	SD	Mean	SD	
CRC worry	11.27	3.17	13.11	3.98	9.62	2.22	.012
Perceived risk (0–100%)	47.33	14.86	44.64	24.42	55.38	27.87	.603
Perceived preventability (0–100%)	46.67	21.27	53.57	25.71	54.62	32.05	.681
Perceive risk (each item)							
I think the risk of CRC is high	3.13	1.19	3.32	0.91	3.08	1.19	.690
Compared with average women of the same age, morbidity of CRC is high	2.67	1.05	3.14	0.89	2.62	1.04	.114
Compared with average men of the same age, morbidity of CRC is low^a^	3.47	0.99	3.11	0.99	3.31	1.18	.503
Recovery from CRC is easier compared with other diseases	3.00	0.85	3.32	1.28	2.38	0.87	.046
If I suffer from colon cancer, I will not completely heal from it	2.73	0.88	2.93	0.81	2.23	0.83	.078

^a^The item's score was reversed before analysis.

## Discussion

Although only 55% of CRC survivors were found to undergo recommended follow-up colonoscopic examinations (Salloum et al., 2012), little is known about the psychological factors underlying surveillance behavior. This prospective study is apparently the first to investigate the predictors of repeat colonoscopy after one year in a clinical setting.

Importantly, we found that none of the psychological factors assessed was associated with colonoscopy after one year. In contrast to a previous study (Salz et al., 2009), we found that cancer threat-related variables were not significantly associated with repeat colonoscopy. The first reason relates to differences in the participants. Salz et al. (2009) studied subjects who were Stage 1–3 CRC survivors. The authors found that many CRC survivors were afraid of CRC recurrence and the authors suspected an association between the perceived risk of recurrence and the degree of intention to be re-examined. All of the subjects in our study had benign tumors and no cancer and this fact may have led to participants deciding not to get screened one year later even though the risk was estimated as high at baseline. Surveillance behavior differs greatly from screening behavior, regardless of whether patients know their test results and health status. Longitudinal studies have found that cancer threat-related variables were not associated with screening behavior (Hirai et al., 2013; Vernon, Myers, Tilley, & Li, 2001). These previous studies showed that cancer threat-related variables were one of the factors motivating surveillance behavior; however, these variables are not considered predictive factors longitudinally. Our study showed that perceived risk and CRC worry were not predictors of follow-up colonoscopy based on factors related to study subjects or study design.

We also examined whether preventability of CRC predicts repeat colonoscopy after one year. The efficacy of execution of behavior or positive expectancy was found to predict the development and maintenance of behaviors. Conversely, these positive variables or optimism had a negative influence on behaviors preventing physical disease (Baumeister, Bratslavsky, Finkenauer, & Vohs, 2001; Stice, Presnell, Shaw, & Rohde, 2005). Because self-efficacy was not significantly associated with screening intention (Salz et al., 2009), preventability of CRC was not likely to predict surveillance behavior. Finally, we found that CRC knowledge was not significantly associated with surveillance behavior. A large survey in Asia found differences among countries in level of CRC knowledge (Wong et al., 2013), suggesting that disseminating knowledge about CRC is important. Nevertheless, CRC knowledge alone did not influence CRC screening behavior (Weinberg et al., 2009).

In contrast, we found that higher educational status and the result of colonoscopy were factors associated with colonoscopy after one year. With respect to the predictive factors for CRC surveillance, surprisingly, educational factors have not previously been studied as a variable (Rolnick et al., 2005; Rulyak et al., 2004). Therefore, our study may be the first to indicate an association between educational factors and surveillance behavior of colonoscopy. However, previous studies have indicated that higher educational status is associated with health behavior (Lantz et al., 1998), low cancer-related death rate (Albano et al., 2007), and a tendency to get screened for CRC (Subramanian et al., 2004). With these studies in mind, we considered it reasonable that educational status is a predictive factor.

We also observed a significant association in the not polypectomy/remaining polyps group between the results of baseline colonoscopy and repeat colonoscopy after one year. These colonoscopic results were considered triggers reminding patients of objective CRC risk. A longitudinal study of breast cancer worry found that patients with a family history of breast cancer perceived a higher risk of breast cancer, with this worry associated with their screening behavior (McCaul, Branstetter, O'Donnell, Jacobson, & Quinlan, 1998). Although psychological factors were not examined, Rulyak et al. (2004) found that the location of the tumor was a rough predictive factor of surveillance behavior. In our study, cancer threat confirmed by the results of baseline colonoscopy may have motivated these patients to undergo repeat colonoscopy.

To examine whether cancer threat affects the relationship between the result of colonoscopy and undergoing repeat colonoscopy, the cancer threat at the time of follow-up colonoscopy is required. Because we did not have this data, we examined the relationship using the baseline psychological data, considering time limitation that is the passage of time affecting patients' CRC threat. Our results showed that cancer threat-related psychological variables differed significantly among our three groups of patients. However, the group with not polypectomy and remaining polyps, with the significant association in the repeat colonoscopy, had the lowest scores on CRC threat-related variables. Also, the group with the highest perceived CRC threat was that of patients with polypectomy and remaining polyps, which had only the second highest rate for repeat colonoscopy. A longitudinal study showing a negative association between perceived risk and history of polyps suggested that screening for CRC may reduce its perceived risk (Vernon et al., 2001). Furthermore, a correct understanding of the results of CRC screening or objective risk was found to influence the perceived risk of CRC (Stark, Bertone-Johnson, Costanza, & Stoddard, 2006). According to both studies, cancer threat-related variables are affected by experience of treatment or a correct understanding of test results.

The relationship between cancer threat and surveillance behavior is inferred from the results of the previous and our studies, as individuals with polyps not polypectomy and remaining polyps were told that no polyps needed to be removed from their colons, although small polyps still remained. Thus, the threat of CRC induced by undergoing a colonoscopy may not be strong, but the necessity of repeat colonoscopy is clear, resulting in a significant relationship with repeat colonoscopy. In contrast, the CRC threat in participants with polypectomy and remaining polyps may be due to the need to remove polyps from their colons and the uncertainty about the status of the remaining polyps. Fear of CRC tests was shown to be a barrier to CRC screening (Jones, Devers, Kuzel, & Woolf, 2010), and cancer threat-related variables also play a functional role in avoidance behavior (Bandura & Menlove, 1968). Consequently, the surveillance behavior of patients with polypectomy and remaining polyps may be more inhibited than in those with polyps/not polypectomy and remaining. Cancer worry may either facilitate or inhibit health behavior, and threat-related variables such as cancer worry and fear could predict health behavior only when beliefs about the ability to perform the behavior and the effectiveness of the protective measures were high. Moreover, the curvilinear relationship between cancer threat and cancer preventive behavior has not been widely tested (Hay, Buckley, & Ostroff, 2005). We found that, in addition to objective risks such as remaining polyps, test results and the efficacy of colonoscopy influenced surveillance behavior. Above all, this longitudinal study was worthwhile in showing a curvilinear relationship between cancer threat and surveillance behavior.

Although the Japan Polyp Study, which was designed to determine the impact of surveillance colonoscopy, is ongoing (Saito et al., 2010), present Japanese guidelines for colonoscopy do not include defined criteria for the resection of polyps <5 mm in diameter (Sano et al., 2004). Rather, the decision to resect is at the discretion of each physician. Thus, some polyps may be removed with others left in place. Informing a patient that some polyps are still present may therefore motivate patients to undergo re-examination. In contrast, informing patients that all polyps had been removed may result in avoidance of reexamination. The latter patients may be encouraged to return for follow-up colonoscopy by being informed of the curability of CRC, including the effectiveness of colonoscopy in reducing CRC risk. CRC threat may therefore both motivate and inhibit periodic surveillance, depending on the situation, making CRC threat a psychological variable likely to affect surveillance behavior. Psychological distress and coping ability may affect the ability of patients to process risk information (Vernon et al., 2001), an association that requires future study.

This study had several limitations. First, most participants were recruited in a single hospital and the sample size was small. Future studies should involve larger numbers of patients undergoing colonoscopy at many centers to minimize sampling bias. Second, the colonoscopist involved in this study knew its purpose. Thus, observer bias may have affected the outcome of this study, suggesting the need for studies performed by blinded colonoscopists.

Despite these limitations, this longitudinal study is considered the first study to identify the predictors of repeat colonoscopy among patients undergoing colonoscopy in Japan. We did not identify psychological factors as predictive factors for colonoscopy use after one year, but educational status and the result of colonoscopy were predictive factors. We also identified a non-linear relationship between high CRC threat and inhibition of screening behavior based on our finding that the CRC threat functions as motivation for the surveillance behavior of colonoscopy. Communication strategies based on these results may improve the rate of re-examination among Japanese patients.
